# Fluid Therapy from Friend to Foe

**DOI:** 10.2478/jccm-2023-0019

**Published:** 2023-07-31

**Authors:** Raluca Fodor

**Affiliations:** Department of Anesthesiology and Intensive Care, George Emil Palade University of Medicine, Pharmacy, Science, and Technology of Targu Mures, Romania

The general management of shock, or any syndrome characterized by inadequate tissue perfusion involves identifying and addressing the underlying cause while also reversing the disorder produced in any of the 4 components of the cardiovascular system (blood and fluid compartment, vascular system, heart and circulatory system) [1] through combined therapeutic methods that are based on rapid volume resuscitation, usually pursuing target goals. Although the “salvage, optimization, stabilization, de-escalation” mnemonic has been utilized as a broad framework for volume resuscitation since 2016 [2], the approach to fluid therapy in critically ill patients remains inconsistent with current clinical practice, contingent upon the practices adopted by individual healthcare institutions.

Given that there is no ideal initial rate of volume repletion, many clinicians rely on recommended volume re-equilibration rates in sepsis. The Early Goal-Directed Therapy protocol involves [3] the use of intravenous fluids - adapted to the patient's weight only-, within 1 hour, vasopressors, and blood transfusions to maintain specific static targets. However, subsequent studies have shown that this protocol may not be superior to standard fluid resuscitation in improving outcomes.

In recent years, there has been growing interest in individualized fluid resuscitation [4, 5], where the fluid choice and amount administered are tailored to the patient's individual characteristics and response to therapy [6] and such practice was associated with reduced mortality, ICU length of stay, and duration of mechanical ventilation [7].

In general, crystalloids are recommended over colloid-containing solutions in patients with severe volume depletion. Normal saline is the most often utilized crystalloid solution for first repletion because data have failed to show consistent superiority of buffered crystalloids over saline, especially when amounts of 2 L are supplied [8]. For large volume resuscitation, saline solution can be given, but the serum chloride levels should be monitored and the administration of saline should be stopped when hyperchloremia develops. Balanced solutions should be reserved for patients who have already received large volumes and in whom the chloremia is rising [8,9,10].

Colloidal solutions, even if they produce a faster plasma volume expansion compared to the administration of crystalloids, numerous randomized trials have failed to demonstrate consistent clinical benefits derived from this advantage and are rarely used as first line treatment in volume resuscitation [11]. The benefit of albumin administration on mortality of patients with shock have not been well-established. Some clinicians still recommend albumin in those with a limited response to crystalloids, on the assumption that albumin might limit the dilutional hypoalbuminemia that usually occurs after isotonic crystalloids, and might protect against pulmonary edema, although clinical evidence supporting these indications are limited [12].

Frequently, following the initial salvage phase, the promptness of preload responsiveness may diminish rapidly [5]. Subsequently, patients often receive fluid administration in a liberal manner, without proper consideration of the patient's fluid status. As too much fluid of any kind can be toxic, the primary objective is to reestablish equilibrium between oxygen requirements and supply [13], all the while reducing the adverse consequences associated with the administration of an excessive amount of fluid.

The novel techniques, many of which use complex imaging technology and computer algorithm [14] to assess the responsiveness to intravenous fluid administration, aim at overcoming the deficiencies associated with static parameters such as blood pressure or urine output, or traditionally invasive tools such as central venous and pulmonary artery catheters and are based on the well-known principle of heart-lung interaction in the ventilated patient and/or visual assessment of cardiac and large vessel blood flow. Depending on the presence or absence of mechanical ventilation and the monitoring in place, this approach involves using dynamic tests [7, 15] like the tidal volume challenge, the passive leg raising test and the mini-fluid challenge performed by measuring changes in pulse pressure variation, stroke volume variation, or oximetric waveform variation in order to assess ongoing fluid requirements. Although not traditionally considered a monitoring technique, heart and lung ultrasound is an important method of evaluating the hemodynamically compromised patient. The ultrasound techniques used to estimate the volume status include: the assessment of the vena cava, pulmonary ultrasound, femoral vein diameter, and doppler of the portal, hepatic or renal veins, but they require training and the images are operator dependent [14].

In compensated shock, blood pressure and cardiac output can be normal even in the presence of altered perfusion at the tissue level, which led to the identification of devices capable of determining the signs of shock at the tissue level. A method of assessing tissue perfusion is the measurement of blood flow in the microcirculation. Despite the vast interest in measurement of the microcirculation, monitoring techniques are still mostly experimental and difficult to implement in clinical practice [16].

In critically ill patients with vasodilatory shock not responsive to volume resuscitation, vasopressors are administered and up-titrated, often to toxic levels, to achieve a specified mean arterial pressure [17]. Since the development of human genome sequencing technologies, common genetic variation for many drug targets, have been identified and probably contribute to interindividual variability [18] in drug and dose-related responses interfering with the individual cardiovascular response to the use of vasopressors also [19]. The clinical outcomes for patients receiving treatment in the ICU may be significantly improved by incorporating pharmacogenomics or the patient's genetic information [20].

Once a patient has been stabilized, efforts should start to concentrate on removing excess fluid by the means of loop diuretics and hemofiltration/hemodiafiltration.

In conclusion, fluid replacement is a vital component of shock management, and the choice of fluid and amount administered should be tailored to the patient's individual needs. The use of dynamic parameters to guide fluid administration holds promise in improving patient outcomes, and further research is needed to determine its clinical utility.
